# Promising Role of Polylactic Acid as an Ingenious Biomaterial in Scaffolds, Drug Delivery, Tissue Engineering, and Medical Implants: Research Developments, and Prospective Applications

**DOI:** 10.3390/molecules28020485

**Published:** 2023-01-04

**Authors:** Lalit Ranakoti, Brijesh Gangil, Prabhakar Bhandari, Tej Singh, Shubham Sharma, Jujhar Singh, Sunpreet Singh

**Affiliations:** 1 Department of Mechanical Engineering, Graphic Era Deemed to be University, Dehradun 248002, Uttarakhand, India; 2 Mechanical Engineering Department, SOET, HNB Garhwal University, Srinagar 246174, Uttarakhand, India; 3 Mechanical Engineering Department, SOET, K. R. Mangalam University, Gurgaon 122103, Haryana, India; 4 Savaria Institute of Technology, Eötvös Loránd University, 9700 Szombathely, Hungary; 5 Mechanical Engineering Department, University Center for Research and Development, Chandigarh University, Mohali 140413, Punjab, India; 6 School of Mechanical and Automotive Engineering, Qingdao University of Technology, Qingdao 266520, China; 7 Department of Mechanical Engineering, IK Gujral Punjab Technical University, Kapurthala 144603, Punjab, India; 8 Department of Mechanical Engineering, National University of Singapore, Singapore 117575, Singapore

**Keywords:** poly lactic acid, human implant, biomaterial, drug delivery, implantation, tissue engineering

## Abstract

In the present scenario, the research is now being focused on the naturally occurring polymers that can gradually replace the existing synthetic polymers for the development of bio composites having applications in medical surgeries and human implants. With promising mechanical properties and bio compatibility with human tissues, poly lactic acid (PLA) is now being viewed as a future bio material. In order to examine the applicability of PLA in human implants, the current article sheds light on the synthesis of PLA and its various copolymers used to alter its physical and mechanical properties. In the latter half, various processes used for the fabrication of biomaterials are discussed in detail. Finally, biomaterials that are currently in use in the field of biomedical (Scaffolding, drug delivery, tissue engineering, medical implants, derma, cosmetics, medical surgeries, and human implants) are represented with respective advantages in the sphere of biomaterials.

## 1. Introduction

Poly lactic acid (PLA) is a thermoplastic polymer derived from various natural resources such as corn starch, sugarcane, biomass, and other vegetable wastes by the process of fermentation. It was first discovered in the 1920s by Wallace Carothers and commercialized in the 1990s at a larger scale owing to its better physical, mechanical, and thermal characteristics. Today, it has made a decent place in various food processing, textile, agriculture, and cosmetic sectors [1,2,3]. Apart from these sectors, its presence can also be felt in mechanically driven plastic equipment used on daily basis. The scope of PLA further widens due to its biodegradable nature particularly, in the field of medicine and in instruments and surgery [4,5]. The medical devices made of PLA are increasing rapidly in numbers due to their desired physical and mechanical properties and will hopefully rise in near future. The most important role which is currently being played by PLA is in medical surgeries and is also the topic of discussion in the present articles. Due to many unfortunate reasons, medical surgeries are performed. These medical surgeries require pre-surgery medications and implant material or devices [6,7]. Due to its desirable characteristics such as compatibility with human tissues, biodegradability, stiffness, non-toxicity, durability, and ease to resorb, PLA is now being considered one of the best-suited biomaterials. However, its applications are restrained due to its low strength, low heat resistance, and difficulty in machining. These characteristics can be improved by bringing various physical and chemical changes in the PLA. For example, blending PLA with other copolymers can bring significant changes in the structure of PLA with improved strength, toughness, and thermal properties, which can be used as a bone implantable material [8]. The strength of PLA can also be improved by reinforcing it with carbon fiber or other synthetic fiber but at the cost of biodegradability and resorbability. Using natural fiber in place of synthetic fiber, the biodegradable nature of PLA can be maintained but decreases the mechanical performance when compared with synthetic fiber. Several research works have been carried out in the past dealing with the physical, mechanical, and compatibility nature of PLA to make it better and better over a period of time for its implementation in medical surgeries and implants [9].

Poly lactic acid in medical implants is being used in many forms such as film, sphere, hydrogels, foam, blends, fiber, particulates, capsules, etc. Its composite offers the ability to embrace surgical applications such as the malfunctioning of tissue or cell in in vitro or in vivo surgeries like the malfunctioning of tissue or cell in in vitro or in vivo surgeries. However, work is still to be done for its improved functioning, longevity, and durability, which cannot be accomplished without past background information and suggestive measures that need to be taken care of [10,11]. The present article, therefore, provides a detailed review of the synthesis of PLA, its behavior with copolymers upon blending, its fabrication for medical devices, and its current application in the medical field. The discussion carried out in this article will not only provide valuable evidence about PLA but also frame a comprehensive background to project PLA as a promising biomaterial. The article also discusses several issues regarding its compatibility with other polymers and with human tissues as a medical implant.

## 2. Poly Lactic Acid and Its Synthesis

The starting substrate to produce PLA is lactic acid (LA), which is basically acidic in nature and at its core structure, the carbon atoms are present in an asymmetrical form. Lactic acid exists in two isomers viz: (i) levorotatory form (D or R (−) lactic acid) and (ii) dextrorotatory form (L or S (+) lactic acid) [12]. D-Lactic acid is extracted from the muscles of animals while L-LA is produced by the fermentation of sugar through the action of bacteria. Beans, peas, corn, sugar beet, soymilk, and potato are some of the chief sources of L-LA. In biomedical applications, L-LA is preferred over D-LA due to several disadvantages such as metabolism rate, synthesis, and lack of optical purity [13]. Apart from PLA, several chemicals, e.g., propylene glycol, acrylic acid, and acetaldehyde are also synthesized from LA [14]. Lactic acid does not possess any charge and, it is very small in molecular size which enables it to infuse in the lipid membranes of cells [15]. It can serve as an energy source and provide antioxidant characteristics that protect from cell damage upon reaching the core of the cell via a monocarboxylate transporter. A large portion of Lactic acid is processed chemically by fermentation in the presence of bacteria whereas a small part of LA is obtained by the process of hydrolysis [16]. Usually, the process of fermentation yields a racemic mixture of LA, L-LA, and D-LA with optical purity [17]. The percentage of L and D in the LA depends upon the strain selected for the process. The biological advantage of lactic acid is not one but many. It is biocompatible with human tissue, provides support to cell tissues, allows accelerating growth of cell generation, and gets absorbs easily if the need arises [18].

**Figure 1:**
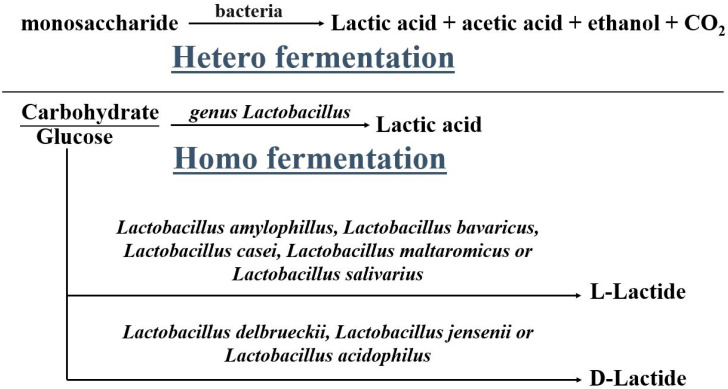
Fermentation of lactic acid.

The production of LA is carried out either by hetero fermentation or homo fermentation in the presence of various catalysts. The isomers formed during the process depend on the enzymes used [19] as shown in Figure 1. The input sources such as glucose, sugar, carbohydrates, etc. used in the production are very costly but can be brought down by using alternate sources such as agriculture waste, biomass residue, and waste collected from the food industry provided the chemical process and the enzymes used are different from the conventional ones [20].

**Figure 2:**
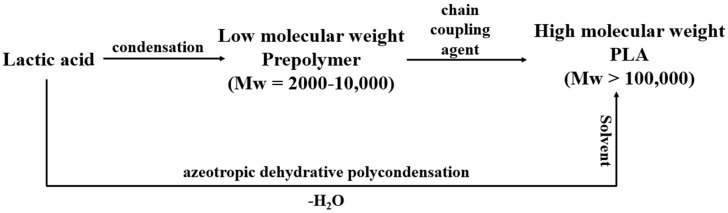
Direct and azeotropic poly condensation.

Formation of Poly lactic acid is a follow-up process performed after the formation of lactic acid. It is carried out via three processes namely (i) direct poly condensation, (ii) azeotropic dehydrative condensation, and (iii) ring opening polymerization. In direct poly condensation, as depicted in Figure 2, LA is dehydrated into oligomers. These oligomers are further dehydrated in the presence of chain coupling agents to form PLA. The whole process is executed without any delay since the chances of degradation of polymer compounds are higher [21]. This process is quite easy to perform but rarely preferred due to the entrapment of moisture in the viscous polymer melt leading to a decrease in the overall molecular weight of the polymer.

In order to produce PLA of high molecular weight, azeotropic dehydrative condensation is performed. It is a time-consuming process and requires solvents of higher boiling points for the removal of dissociative molecules of water from the PLA compound. Longer reaction times and the use of inorganic solvents make this process very unsuitable and uneconomical for large production [22]. Another way of converting LA into PLA is ring-opening polymerization (ROP). In this process, the dehydrated and condensed LA is converted to oligomers under high temperature and low pressure thus releasing excess moisture from the small chain molecule of PLA. These small chains of PLA are termed meso-lactides. Thereafter, depolymerization of meso-lactides at a temperature higher than the melting point of lactides but lower than the degradation temperature of PLA is performed, resulting in the yield of poly lactic acid of high optical purity [23]. The PLA extracted in ROP is converted into pallets after the crystallization of the liquid resin.

### 2.1. Copolymerization of PLA

Despite being the most influential polymer for biomedical devices, a large scope for improvement still exists in various properties of PLA such as degradation characteristics, improved biocompatibility, rheological characteristics, crystallinity, and mechanical strength. These properties can be enhanced by the copolymerization of PLA with several other biocompatible polymers. With the help of copolymerization, PLA can be used in a wider range of biomedical applications. The advantages of various copolymerizations of PLA have been tabulated in Table 1.

### 2.2. Fabrication of PLA Composites for Biomedical Applications

Manufacturing of PLA-based composite particularly for medical implants has picked up a noticeable pace due to a continuous increase in demand [45]. Various techniques have evolved over the period such as freeze drying, melt blending, solvent casting, bioprinting, electrospinning, Supercritical fluid (SCF) technology, etc. with respective merits and demerits and have shown their potential of manufacturing bio material efficiently [46]. These processes work at different temperatures and pressures. In some, an organic solvent is used to achieve the required pattern at the surface while in others, composites are soaked in a solution to obtain a uniform structure throughout the volume [47]. The changes brought in the PLA matrix for the making of biomaterials are discussed for the following processes: freeze drying, electrospinning, SFC, and solvent casting.

**Figure 3:**
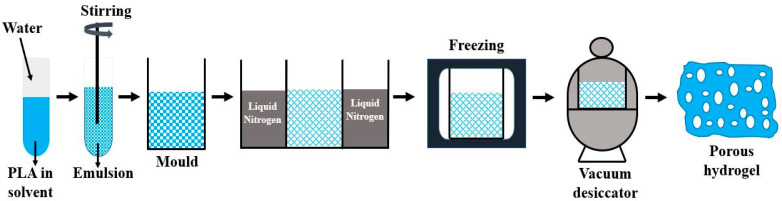
Freeze drying technique for making porous hydrogels.

**Figure 4:**
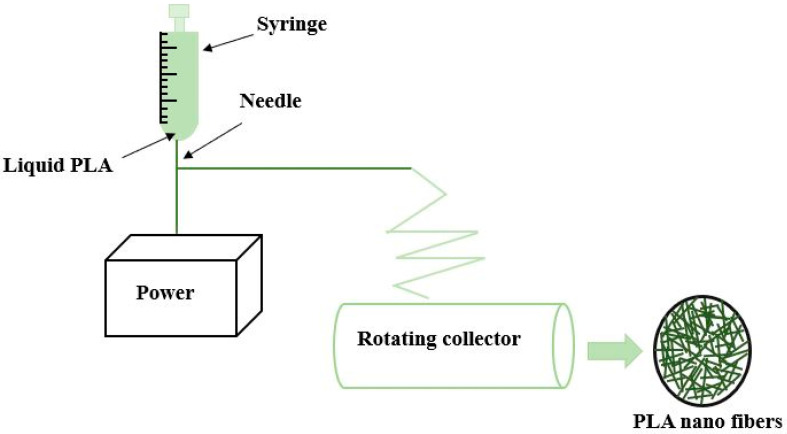
Electrospinning method for producing nano fiber.

**Figure 5:**
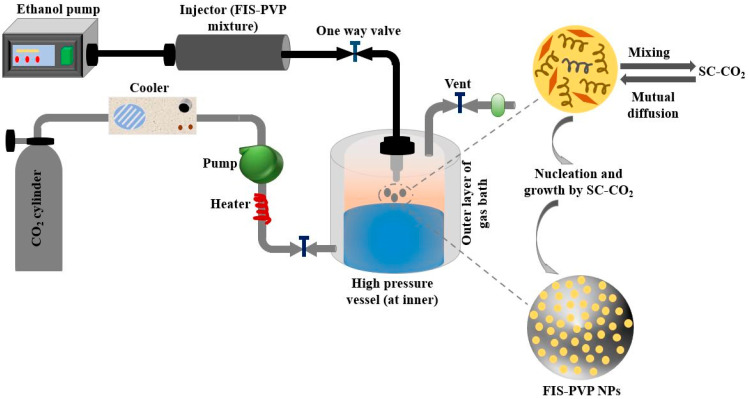
Schematic illustration of super critical fluid CO_2_ method for preparing Fisetin-Encapsulated Poly (Vinyl Pyrrolidone) Nano particles.

**Figure 6:**
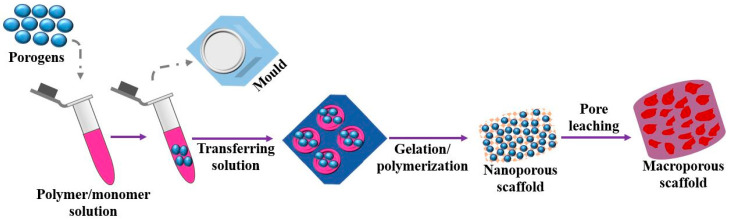
Solvent casting method for fabricating macro porous scaffold.

(a) PLA scaffolds for tissue engineering can be efficiently prepared by the method of freeze drying. Liquid PLA is dissolved in water and undergoes stirring to achieve a homogeneous polymeric solution. This solution is cast in a mold along with cooling at freezing temperature. Ice crystal is then formed at the interstitial sites, which leads to the aggregation of polymeric molecules. A vacuum desiccator is used to evaporate the solvent through sublimation forming the porous hydrogel having interconnected pores as shown in Figure 3. The hydrogel obtained in the process is highly porous, which can be altered by controlling parameters such as freezing temperature, concentration of solution, size of ice crystal, and Ph value of solution. This hydrogel is readily applied for cell seeding in tissue engineering. The degradation rate and solubility of the hydrogel can also be modified by its cross-linking with ultraviolet radiation and treatment with citric acid, carbodiimide, and glutaraldehyde [48]. This process limits its application for scaffold fabrication due to the presence of harmful residue, consumption of high energy, and very long hours of dehydration [49].

(b) To produce fibers ranging from tens of micrometers to nanometers, electrospinning is used as depicted in Figure 4. A highly concentrated solution of PLA in melt form under the influence of a strong electric field is pushed through a fine needle and collected at the rotating cylinder for the production of continuous fiber [50]. The fiber characteristics such as strength, porosity, texture, surface area, etc. are controlled by maintaining the rate of flow through the syringe and applied voltage. Nano fibers produced in this process exhibit exclusive characteristics such as a large surface-to-volume ratio, high porosity, and light weight. The native collagen fibrils found in the extracellular matrix are remarkably intimated by the morphology of nanofiber produced by electrospinning. In addition, exchanging of nutrients and gaseous are also possible provided the macropores at the scaffolds should be maintained. For the purpose of regenerative medicine, electrospinning is mostly preferred over other processes due to its ease of fabrication and flexibility. Electrospun fibers have the capability to mimic the characteristics of the extracellular matrix but the use of toxic solvents limits the horizon of this process. Insufficient cell infiltration and inhomogeneous cell distribution are other demerits of this process. Apart from PLA, various other polymers such as polyurethane, polycaprolactone, nylon-6 poly (glycolic acid), etc. can also be electrospun [51].

(c) Another way of making bio material (3-D scaffolds) for tissue engineering without using solvents is Super critical fluid (SFC) as represented in Figure 5. The advantage of using supercritical fluid is its control over architectural activities of internal porosity, elimination of solvent, and mimicking the growth of bioactive material at the surface [52]. The coexistence of liquid and gas makes it an exclusive method as compared to other methods that exist in either liquid or gas form at normal temperature and pressure. Till now, the most widely used supercritical fluid is carbon dioxide due to its favorable characteristics such as availability, user-friendliness, low cost, and non-toxic behavior. Due to its low critical conditions, CO_2_ is extensively used to process thermal sensitive/biological compounds. This technique is highly versatile that various biopolymers like PLLA, PGLA, forms of poly saccharides, and proteins can be easily processed for the fabrication of very highly porous scaffolds [53]. In an experimental analysis, it was observed that 95% seeding efficiency with a viability of 40% was obtained in a single day of culturing in the rats [54]. Apart from the above-said advantages, the control of particle size and morphology is very difficult in this process, which happens due to polymer solubility in super critical fluid.

(d) One of the simplest techniques of processing scaffolds for tissue engineering is solvent casting as shown in Figure 6, which does not require any specific equipment. Here, the liquid PLA is dissolved in a solvent followed by the addition of salt of a specific size in the solution to make it homogenous. The mixture obtained is molded in a 3-D through casting. The solvent is made to evaporate from the solution leading to the formation of a matrix with uniform distribution of particles of salt. Afterward, water is added to the polymer matrix to filter out the salt particles from the matrix resulting in the formation of a porous structure capable of seeding cells [55]. The selection of solvent is the crucial parameter that decides the final surface texture of the matrix such as heterogeneity, the orientation of crystals, swelling parameter, and physical characteristics like deformation rate. These characteristics will decide the specific domain where the produced scaffold is used. This process has several advantages such as short processing time and low maintenance cost, and it can produce scaffolds of different porosity by varying the size of the porogens [56]. Various biopolymers such as PLLA and PGLA can be easily processed by this technique. The chief advantage of this method is pore size and its porosity can be easily modulated. In addition, drug incorporation becomes easier within the scaffolds. Besides having such attractive characteristics, solvent casting is very often used for making scaffolds due to the use of harmful solvents in the solution [57].

## 3. Scope of PLA in Biomedical Applications

**Figure 7:**
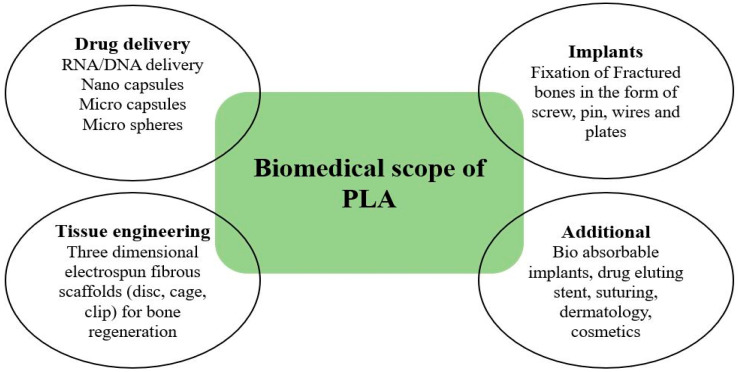
Biomedical applications of polylactic acid.

Polylactic acid has a notable reputation in many biomedical applications due to its bioresorbability and biocompatibility with human tissues. Scaffolding, drug delivery, medical implants, suturing, membrane covering, derma, cosmetics, etc., are some of the major fields of biomedical, as depicted in Figure 7, where the role of PLA has been appreciable in the last decade [58]. Physical and chemical modification of PLA is required to render its service in a diversified form in medical applications. Sometimes, surface modifications of PLA, such as plasma treatment, radiation-induced technique, etc., are carried out for enhancing the biocompatibility of PLA. Blending of PLA with copolymer is also performed to achieve the desired behavior. Polydioxanone (PDS), Polyglycolide (PGA), and PLA are the most common biopolymers used extensively in biomedical applications [59], but due to favorable properties, more attention is being given to PLA as a biomaterial.

### 3.1. Polylactic Acid in Drug Delivery

**Figure 8:**
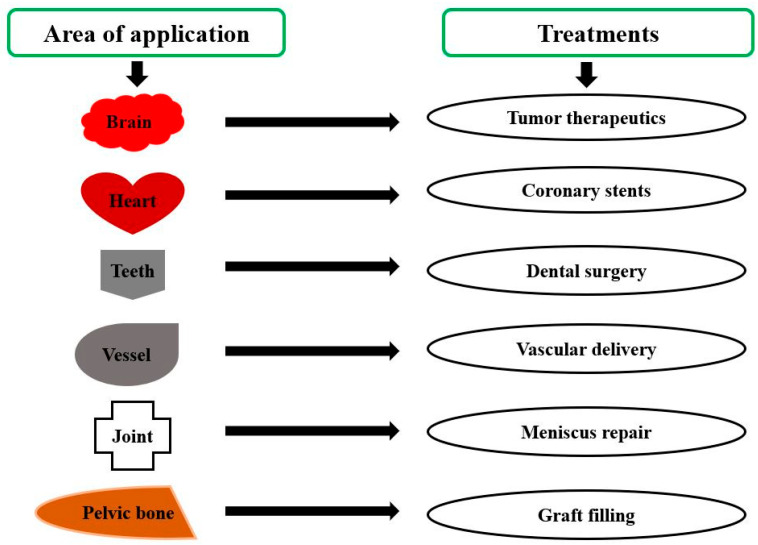
Poly lactic acid-based drug delivery systems (Adapted from the reference [64]).

Developing a drug delivery system (DDS) to address new challenges is only possible through innovative approaches. Supplying the drug to various parts of the human body at a specific quantity with maximum therapeutic potential and minimum side effects has always been a longstanding desire of medical practitioners [60]. Various systems have been evolved as drug carriers, for example, nano capsules (NC), nanospheres (NS), liquid crystal, multifunctional dendritic polymers, vesicles, micelles, etc., but still, the oral route is restricted to small molecules like NC or NS [61]. Some typical DDS based on PLA are represented in Figure 8. Drug delivery based on PLA is showing promising results for diagnosis, therapy, and imaging. For instance, micro and nano particles (NPs) delivery systems produced by the blends of methoxypoly (ethylene glycol)/PLA (mPEG/PLA) are being considered as a good replacement for conventional Kolliphor EL [62]. A range of properties of the blend, like drug loading, particle size, release profile, etc., can be easily controlled by varying the percentage of mPEG/PLA in the blend [63].

PLA can be transformed into micro capsules (MCs), dosage, pallets, and NPs due to its biodegradability, biocompatibility, strength, and solubility in various solvents [65]. Several sustainable drugs like protein/peptide and DNA/RNA for delivery purposes can be made from modified and unmodified microparticles (MPs) and NPs of PLA [66]. MPs and NPs are very small particles that enable them to permeate through biological barriers, especially blood barriers of the brain. Polylactic acid-based MCs are extensively applied in the delivery of drugs for prolonged administration in a large variety of medical agents [67,68,69], such as local anesthetics and vaccines, contraceptives, and antagonists.

Peptides and proteins-based PLA DDS are designed for specific purposes and receive significant attention due to their effectiveness even at comparatively lower dosages [70]. Reconstructive surgery of the face can be carried out by temporary filling of the microsphere of PLLA. Transcatheter arterial embolization, an efficient procedure for managing hemorrhages, tumors, and fistula malformation, can be healed with embolic material composed of microsphere of PLLA [71]. It has been seen that PLA-based bio materials lack cell interaction due to the deficiency in chemical functionality, which also restricts the continuous release of hydrophilic molecules, especially proteins [72,73,74]. These limitations can be overcome by introducing an amine functional group in the PLA-based DDS and additives fabricated by direct conjugation. PLA-based nanomaterials are also used for stimuli response. These stimuli responses effectively target delivery where DDS acts as an active member rather than a passive carrier [75,76,77].

Nanofibers of PLA-poly butylene adipate (PBA) blends are very effective for the transdermal delivery of teriflunomide (an antirheumatic agent). These nanofibers quickly degrade and dissolve in the cells [78]. The PLA particulate system is important for stimuli and targeted DDS because it can target decayed cells [79]. The synchronization of stimuli-responsive PLA nanocarriers with pathology by in vivo hydrogelation is helpful in the healing of diseases such as tumors by maintaining the pH value of extracellular tissues [80,81,82]. PLA can also be synthesized to design dendritic core shells for enhanced transport capacity by mixing Polyethylene glycol (PEG) monomethyl ether shells in PLA blend through imine bonding [16].

Films based on PLA have been extensively explored for drug delivery systems [83]. Paclitaxel-eluting stents were developed by blending ethylene-vinyl acetate copolymer (EVA) with PLA and examined the effect of varying the ratio of EVA and PLA in the blend on the rate of drug release [84]. It was reported that the ratio of EVA and PLA in the blend has a significant effect on the rate of drug release. Films for wound dressing applications for the drug release of metronidazole and gentamicin sulfate are also being developed by the blend of PLA and PEG. Nanoparticles based on PLA with monoclonal anti-bodies (mAb) characteristics are being developed for the drug delivery of specific antigens on breast epithelial cancer cell lines [85]. Both small and large molecules can be efficiently targeted to the cell or tissue of interest.

### 3.2. Polylactic Acid in Implants

Several metals such as titanium and its alloys, stainless steel, cobalt, and chromium have been implanted in the human body in the forms of plates, pins, screws, and wires for operating bone fixation [86]. These metals have shown incredible potential in bone fixation surgery in the last 3 to 4 decades. However, a few disadvantages related to metal implants, such as low corrosion, high density, and low biocompatibility, make way for biodegradable polymers, particularly PLA, to replace metal implants [87]. Polylactic acid has sufficient mechanical strength that it can serve as bone implants satisfactorily. Furthermore, it is biodegradable; thus, the chances of side effects in human bones are negligible. The current state of PLA as a bone fixation material has uncovered many possibilities for development in bone implants [88]. By synthesizing the PLA with L-PLA and D-PLA, plates and screws for fracture fixation can be made. For orthopedic applications, films of PLA have been fabricated that showed good compatibility with fibroblast, nasal septum cells, and osteosarcoma cells [89,90].

A blend of PLA and polyvinylpyrrolidone (PVP) for ocular implants is being prepared for the enhanced release rate of fluorometholone delivery [91]. By adding surfactants such as ethylene oxide or propylene oxide in the blend of PDLLA/PLLA, a potential implant for orthopedic and dental applications can be prepared [92]. The commercialization of drug-eluting stents prepared from the blend of PLA is under consideration by several pharma companies [93]. Nanofibers produced from PLA/PCL embedded by 5-fluoro uracil are also used as drug-eluting stents to treat cancer [94,95,96]. The release of cancerous drugs is higher for hybrid nanofibers than pure polymers because of the combined effect of immiscible polymers due to their superior potential in therapeutic implants [97].

The biodegradability of polymer within the human body is one of the most important characteristics a polymer should demonstrate. As far as PLA is concerned, it is used to reconstruct ligaments and tendons due to its high retention characteristics [98]. Stents are also prepared from PLA for urological and vascular surgery [99]. Three-dimensional scaffolds of PLA are extensively made for the gene therapy of diseases like cardiovascular, bone, and cartilage regeneration and to cultivate different types of cells during orthopedic and neurological treatment [100]. Therapeutic implants like bone formation, intramembrane, endochondral ossification, etc., can be performed with the biomaterial prepared by seeding osteogenic stem cells on PLA scaffolds [101].

Plates and screws used in maxillofacial osteosynthetic surgery are made from platinum, but with several disadvantages such as mutagenetic effects, problems in removal and palpability, etc., their extensive implementation is restricted. To resolve these problems, PLA is being put forward as an alternative to performing maxillofacial osteosynthetic surgery in the near future [102].

### 3.3. Polylactic Acid in Tissue Engineering

**Figure 9:**
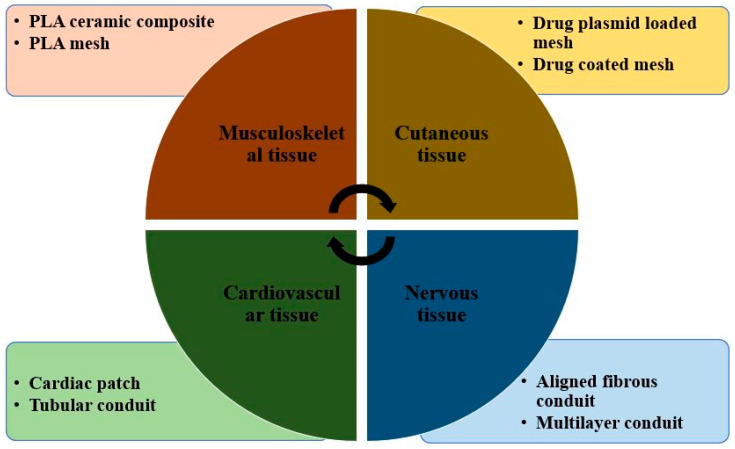
Poly lactic acid-based tissue engineering bio materials [Adapted from the reference [105].

With recent developments in biomedical engineering, PLA-based biomaterials are gradually making their place in tissue engineering as demonstrated in Figure 9. Although numerous promising results were obtained with few metal implants due to the non-biodegradability of metal, biopolymers are now being projected as a potential scaffolds application [103]. In addition, due to their flexibility and tailorable properties, biopolymers find greater scope in tissue engineering than metals. The most common biopolymers used for scaffoldings are PLA, PGA, and PLGA, having approval from the Food and drugs administration [104]. At the same time, constructing scaffolds is the most important thing to be taken care of for the adhesion of cells with polymer. Polylactic acid is hydrophobic and has a slow rate of degradation in the environment of water. Using PLA in scaffolding in its pure form will not be a good idea; therefore, its copolymerization through blending and forming with other biopolymers is usually carried out to enhance its degradability and biocompatibility [105]. In recent years, several techniques have evolved for making porous PLA scaffolds. These are particles leaching, foaming, and electrospinning.

Till now, many PLA-based composites for tissue engineering and scaffolding have been prepared. For instance, PLA-based octadecyl amine composites fabricated via solution preparation technique in the presence of chloroform and nanodiamond showed improved mechanical properties [106]. The basic requirements for the scaffolding and grafting of vascular tissues are antithrombogenicity, application-oriented, sufficient mechanical strength, and bio compatibility to match with the tissue or blood vessels [107]. Presently, matrices based on collagen and hyaluronan are the most prevalent scaffolds applied in clinical applications because they deliver substrates, a necessary element in articular cartilage [108]. Under the trade name of BioSeed-B and BioSeed-C (Bio Tissue Technologies AG, Freiburg, Germany), scaffolds of PGA/PLA, polydioxanone, and polyglactin are also used for the reparation of cartilage.

**Figure 10:**
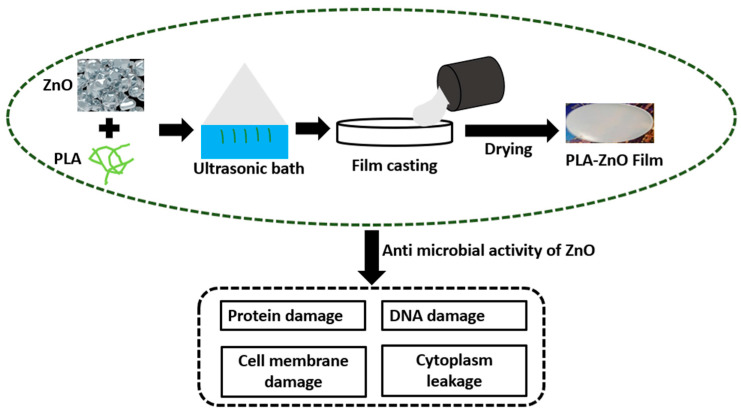
Antimicrobial activity of PLA-ZnO films to eliminate gram negative and gram positive bacteria (Adopted from [122]).

For the regeneration of bone, three-dimensional (3D) fibrous scaffolds are mostly recommended. A 3D micro fibrous scaffold prepared via electrospinning technique followed by mechanical expansion showed huge potential in the proliferation of osteoblast by offering a suitable substrate for bone formation and cell infiltration. For this, an experiment on rabbits was carried out which showed noticeable changes in 2 to 4 weeks [109]. To modulate biodegradability and enhance the compatibility of PLA for tissue growth, recovery, and scaffolds, it can be synthesized with various copolymers. A novel technique for creating tissue scaffolds for bone tissue is 3D printing [110]. Several types of scaffolds made of PLA, such as porous cages, solid disc PLA-filled and coated scaffolds, can be created by 3D printing [111]. PLA-coated scaffolds filled with collagen were tested for biocompatibility and endotoxin production. It was found that the scaffold showed enhanced compatibility with tissue, and the endotoxin level was far below the permissible limit [112]. Printed discs based on PLA have shown rapid growth and expansion of different cells such as endothelial cells, osteoblasts, and osteoblast-like cells. The steady release of stromal-derived factors that helps in the growth of endothelial cells and encourages the formation of neo-vessel can be achieved by cages made of PLA, thus verifying the potential of 3D PLA scaffolds [113]. To increase the rate of vascularization and healthy bioactivity of osteoinductive cells in bone tissue engineering, the biodegradability of 3D scaffolds plays a crucial role. Because of the favorable properties like flexibility, practicality, bioresorbability, and biocompatibility, 3D-printed PLA scaffolds can be used to control the release of deferoxamine necessary for osteogenesis and angiogenesis for the construction and development of fractured bone [114]. Poly hydroxyapatite (PHA)/PLA 3D composite scaffolds have also been explored for inflammation and bone repair. It was observed that PHA/PLA 3D scaffolds showed better compatibility, bioactivity, and osteoinductivity with a low chance of inflammation [115]. To further enhance osteogenic activity in bone tissue engineering, PHA/PLA scaffolds functionalized with citric acid and polyethyleneimine have been developed in the recent past [116]. The successful development of 3D printed PLA/PHA/silk scaffolds clip for the bone support showed improved strength and biocompatibility as compared to conventional clip [117,118,119] is another indication of the potential of PLA in bone tissue engineering. Recently, more attention has been given to regenerative treatments using stem cell therapies. The available reports suggest that Mesenchymal stem/stromal cells are the best-fitting materials in tissue replacement [120]. Poly lactic acid-based bio dental implants are under development. The latest report suggests that silica nano filler reinforced PLA bio material can serve the purpose of dental filling in the tooth cavity. Moreover, the biodegradability and compatibility of the prepared amalgam showed satisfactory results [121]. However, research and development need to be carried out continuously to obtain the best-fitting PLA-based dental implant. Poly lactic acid can efficiently host the ZnO particle to activate the antimicrobial activity for the regeneration of tissue or cell [122]. Figure 10 illustrates the biological activity of PLA-filled ZnO nano particles in which Gram-negative and Gram-positive bacteria are reduced by 99% due to the high interfacial interaction between nano filler and PLA.

Table 2 summarizes some typical composites based on PLA, which are commonly adapted in the form of organs, implants, and scaffolds.

## 4. Food and Drug Administration Approved PLA Formulations Used in Medicine

The use of PLA in various medical surgeries, implants, and drug deliveries is often encountered with several physical and biological issues such as poor retention at delivery sites, poor bio availability, longevity, limited water solubility, etc. [129,130,131]. To overcome these problems, PLA is coupled with various other biocompatible polymers for the fabrication of nano formulations used in medical therapeutic deliveries as approved by the FDA [132,133,134]. For instance, a formulation based on PLGA-b-PEG-b-PLGA of size 77–84 nano meter (nm) has the advantage of sustaining oral formulation and therefore extensively used in oncology [135]. While PLA-PEG nano formulation of size greater than 200 nm has good healing characteristics in healing bacterial infections [136]. Hydrogels of PLA-based formulation also have a significant role in medicinal therapeutics. It has a vital role in birth control gene therapy with the formulation of PLGA-b-.

PEG-b-PLGA [137]. Moreover, nano formulation of size ranging between 200 nm to 300 nm is very much beneficial in the application of oncology [138,139,140]. In the field of vesicle therapy, PLA-b-PEG with a formulation size under 200 nm is highly useful in pH-responsive release systems [23,139,141].

## 5. Current Limitations

Poly lactic acid is more often avoided due to its high price since it is more expensive than petrochemicals. Its method of manufacturing is more intense and its yield is not as good as compared to any other conventional polymer. Its working temperature is very low due to its low melting point and its coblending with another polymer is very hard to carry out. It is highly permeable and thus releases oxygen and water molecule very easily. Moreover, it takes more time to decompose as compared to other bio polymers. Techniques are under development to enhance its decomposition rate and hoping to see an improved PLA with a better decomposition rate.

## 6. Conclusions and Future Scope

The kind of characteristics PLA possesses makes it suitable for bio implants and medical surgeries with the advantage of being processed at a relatively low cost as compared to other polymers. Its compatibility with other polymers to obtain improved mechanical and thermal characteristics widens its scope for the fabrication of medical devices. Poly lactic acid has proved to be a versatile polymer, which gives it an added advantage of being tailored into different forms such as nano fiber, micro capsules, nano particles, and hydrogels. By incorporating various fibers in PLA, a significant improvement in strength can be achieved that in near future can replace metallic material in bone implants. Various favorable properties of PLA such as bioresorbable, biocompatible, good endurance, ease of fabrication, and compostability are signaling toward its higher exploitation in near future for the development of biomaterial implants. The properties enhancement of PLA by blending it with various copolymers is playing a crucial part in expanding its scope in the biomedical domain (Scaffolding, drug delivery, tissue engineering, medical implants, membranes covering, derma, cosmetics, medical surgeries, and human implants). As far as its applications are concerned, PLA is found suitable for drug delivery, implants, and tissue engineering, which is expected to grow in near future with continuous research and developments. Although PLA has several limitations such as various attributes of degradability, strength, ease of processing, etc., it is expected to remain the preferred polymer for the development of biomaterial. However, as far as the literature is concerned, various aspects such as interfacial energy, barriers and optical properties, influence of processing temperature, environment, and storage (aging) on the properties of PLA have not been fully evaluated yet. Therefore, a lot of work is still to be done to make PLA viable as a permanent, cost-effective, and safe solution for its implementation as a biomaterial in the human body.

## Figures and Tables

**Table 1:** Various copolymers with PLA and their properties.

Copolymer	Architecture	Copolymerization Technique	Enhanced Properties	Applications	References
Poly(D,L-lactide-co-glycolide) (PLGA)	Linear polymer	Solution poly condensation, ROP, segmer assembly polymerization	Shortens the degradation time, higher molecular weight of copolymer	Drug delivery system	[24,25,26]
Metal-Centered Star-Shaped PLA (Co)Polymers	Star polymer	atom transfer radical polymerization	Hydrophobicity at core and hydrophilicity at corona	Drug delivery of optical imaging, biomedicine	[27,28,29]
Poly ethylene glycol-PLA	Star polymer	Michael-type addition reaction	Lower degradation time from few days to month, enhanced mechanical strength, and imparts thermal responsive behavior	Scaffolds, tissue engineering, biocompatible hydrogels, and drug delivery	[30,31,32]
Polycarbonate-PLA	Linear polymer	Formed by the addition of hexamethylene diisocyanate in the chemical reaction	Improve crystallinity, rheological behavior, mechanical properties, and higher elongation at break	Medicine, tough membrane for stimuli drug delivery system, and tubular scaffolds	[33,34,35]
Polyhedral oligomeric silsesquioxane-PLA	Star polymer	ROP, solution casting, reversible addition fragmentation transfer (RAFT) polymerization	Enhanced ductility, improved toughness, and elongation at break	Nerve engineering, serve as basis for collagen	[36,37,38]
Poly vinyl alcohol-g-PLA	Graft polymer	Graft polymerization	Crystallinity and biodegradability enhance, melting point increases and glass transition temperature improves	Agriculture and food packaging and drug delivery	[39,40]
PLA-Glycidol	Branch polymer	Terminal ring opening polymerization	Thermal behavior improved; hydrophilicity enhanced.	Biomedical and industrial applications	[41,42,43]
PLA-Co-Polyesters	Graft comb polymer	Graft polymerization	Increase in hydrophilic characteristics and biodegradability	Scaffolding and tissue engineering	[44]

**Table 2:** PLA-based composites for medical.

Manufacturing Technique	Biopolymer Composition	Influencing Properties	Domain of Application	References
Electrospinning	Tricalcium phosphate-Poly(L-lactic-co-glycoside)	Excellent moldability and absorbance ability are enhanced.	Repairs bone defects	[123,124,125,126]
	Gelatin-PLLA	New calcified bone formed within 84 days of induction, improves cell proliferation and adhesion.		
	Silk fiber-PLLA	Enables uniform distribution of cells in the matrix and improves cell adhesion.	Engineer cartilage tissues	
	Collagen-PLL-co-glycolide)	Matrix becomes hydrophobic and enables easy induction of myogenesis.	Regenerate skeletal tissues	
	Collagen1-PDLLA	Hydrophobicity increase, higher rate of cell proliferation, and improves stability.	Bone- reconstruction	
	Collagen-PLLA	Plays significant role in invitro osteogenesis, large bone aggregates are obtained due to the even distribution of minerals, the expressions of osteoblastic genes obtained are comparable and higher.	Bone -regeneration	
Freeze drying	Gelatin-PLA	Decreases inflammation, cell proliferation, and attachment enhanced.	Repair cartilage	[123,124]
SFC	Demineralized bone matrix-PLA	Mechanical strength improves	Repair bone defects	[126]
3D bio printing	PC-poly(L-lactide-co-glycoside)-triphosphate	Capable of forming new bone around implant, Osseo-integration	Bone-reconstruction	[127]
Electrochemical	Silk fibroin-PLA	Cardiomyocytes functionality improves, better swelling characteristics and comprehensive modulus	Application of nursing and regeneration of cardiac tissue	[128]

## Data Availability

Not applicable.
